# Supplementary material for: Patterns of Intron Gain and Loss in Fungi
Source: PLoS Biol. 2004 Nov 30;2(12):e422. doi: 10.1371/journal.pbio.0020422 (PMC532390; doi:10.1371/journal.pbio.0020422)
Supplement: Table S1 — Also available at http://genes.mit.edu/NielsenEtAl/. (4.3 MB ZIP). [file pbio.0020422.st001.zip › NielsenEtAl/html/514.html]

AN9538.1.NCU06503.1.MG00373.1.FG05399.1


```
 CLUSTAL W (1.82) Multiple Sequence Alignments - Introns Inserted


Sequence 1: MG00373.1	795 aa
Sequence 2: FG05399.1	804 aa
Sequence 3: NCU06503.1	808 aa
Sequence 4: AN9538.1	752 aa
Alignment Length: 826 aa
Number Identitical Residues: 365 aa
Alignment Score (without introns) 19213


MG00373.1 	MPAANVAAIPTDA--------ASAPASRKTS--LAPERKYKCQFCNRAFSRSEHRSRHER
NCU06503.1	MPGASVAVAPTTAPATTTPAPTSASTPRKAPGATQPERKYKCQFCARAFSRSEHRSRHER
FG05399.1 	MPGANVAVLPPPA--------ASTASSRKAS--LAPERKYKCQFCNRAFSRSEHRSRHER
AN9538.1  	------------------------------------------------------------
          	                                                            

MG00373.1 	SH1TKER~PFKCMKCRSTFVRRDLLLRHDRTVHAKDGGVPLHSDGKRRGGAK--PRAVAT
NCU06503.1	SH1TKER~PFKCLKCRSTFVRRDLLLRHDRTVHAKDGGIPLHSDGKRRGGPKAAPRPATG
FG05399.1 	SH1TKER~PFKCMKCRSTFVRRDLLLRHDRTVHAKDGGVPLHSDGKRRAGPK--TRAVGA
AN9538.1  	--~MNDP0---CLKCRSTFVRRDLLLRHDRTVHAKDGGVPLVSEGRRRGGGVR-KASSPA
          	    ::     *:*************************:** *:*:**.*      .   

MG00373.1 	PNKEPIGIDAAALEQIEASSEGVFDVETAAMLVADLHHKATAAMRAN--SGEGSSGAAYP
NCU06503.1	PAKTPMDIDTSALEQLEASSDGIFDVETAAMLVADLQHKATSQARANGAPYDGNGTMSFS
FG05399.1 	PSKSSIAIDTSTLEQMESGADAMFDVEAAAMLVADLHHKATAAMREDH-SYEDSPSMAYS
AN9538.1  	PSKPSVTIDPTTLEQIEASSDGMVDLETAAMLMTDFQHKAAAAATGQVHDRAES-DRSFS
          	* * .: **.::***:*:.::.:.*:*:****::*::***::    :      .   ::.

MG00373.1 	QSGANMMDSPVSFPSGPIAIP--QWDTFMPQAVAEPKAHS-------IASSASGSFESQP
NCU06503.1	QTSTPTIEPSVPFTNGALGLP--QWDSFISH---DSKAHS-------VIS-GPGSFDTSH
FG05399.1 	PHNASAMESTVTYPSGAIALPQVQWDGFMSQSVAEPKAHS-------ITSSASGSFESQP
AN9538.1  	PGRGSLLEPPVSYLSGNATLPQMPWDSLVSP--TESKHHLSPFVSQDAASESHGLMDRHV
          	      ::..*.: .*   :*.  ** ::.   ::.* * :.  :..  *.. * ::   

MG00373.1 	NFNG--SLAQHPAQLPPLGTP-TLNA1PAPPHS---PFLAHGGVHSPVESHQAHSMAGFK
NCU06503.1	PNHMAPMGGQHINGLPLSSLPPSVNG~TPTPQS---PFLPHR-THTPVDATQAHAGQKPP
FG05399.1 	SFTSGATMQPQSNQLPPINGQ-NCNG~LVPALQSMINSLPNSAAAPPSPWVSESSKAGYK
AN9538.1  	TDSMAPSLHSLVNSLPVSGNS-TPNA~LSPYPSMTGPVSPVNYRRSPGPSQ---ALTLPK
          	    ..        **  .   . *.   .  .      .     .*       :     

MG00373.1 	APQVIGDEERNMILDNIRTSDSEQAIPDGFRLPNLPSLNRYLATYFGLFHHHLPFLHPSS
NCU06503.1	PPQINSDEERNMILDNIRAHDAEHALPEGFRVPSLPSINRYLATYFGLFHHHLPFLHPAS
FG05399.1 	APEVSGDDERNAILDNIRNADSEHAIPEGFRLPGLGSLNRYLSTYFGLFHHHLPFLHPAS
AN9538.1  	APQIANDLERNQIVERIRLADSLGVLPESFQLPTTAALNKYLTTYFNLYHHHLPFLHQES
          	.*:: .* *** *::.**  *:  .:*:.*::*   ::*:**:***.*:********  *

MG00373.1 	FKPTEVSSPLLLAVLSIGALYAFDQDQAYALHIGSKVLVNQFLQNKENFSSRQCPLWTMQ
NCU06503.1	FEPTRVSPPLLLALLSIGALYAFDQENAYMLHIGSKILVNQFLQHKENFSSRKCPLWTMQ
FG05399.1 	FHPTKVSPQLLLAVLSIGALYAFDQEQAYMLHIGSKVLVNQFLQSKENFSSRKCPLWTMQ
AN9538.1  	FKPTTASSPLLLAVLSIGALYTFERQHAFMLHVGSKMLVNQFLQHKDNFDSRKCPLWAMQ
          	*.** .*. ****:*******:*::::*: **:***:******* *:**.**:****:**

MG00373.1 	SSLLNMIFASWSGDPKGLEWACSIKSLLAN~MVSGNRYELKLRQEAREARLPSRAEWIED
NCU06503.1	SSLLNMIFASWSGDPKGLEWACSIKSLLAN~MVAGNRYELKLRQEAREGRPPTRAEWVED
FG05399.1 	SNLLNMIFASWSGDPKGLEWTCSIKSLLAN~MVAGNRYELKLRQEARTNAKPTRAEWVED
AN9538.1  	STLLNMIFESWSGDPKGLEWTCSIKSLLAN0MVAGNRYQLKLRTEAREGRQPTREEWIED
          	*.****** ***********:********* **:****:**** ***    *:* **:**

MG00373.1 	EGCRRTYYAVYIFFGLLTLTYNHTPAIGFNEFEDLQLPSTEGLWNAVVSDEAAWQEQLKA
NCU06503.1	EGCRRTYYAVYIFFGLLTLTFNHTPAINFNEFEDLQLPATEFLWNVKVADEVAWQKHLEA
FG05399.1 	EGCRRTYYAVYIFFGLLTLTYNHTPAISFSEFEDLQLPSTETLWNLQVADEATWQEHLAA
AN9538.1  	ESCRRTYYAVYIFFGMLTLTFNHTPAMSFDEFDNLELPSSESMWNLDVNDDEAWRRNLAS
          	*.*************:****:*****:.*.**::*:**::* :**  * *: :*:.:* :

MG00373.1 	SHTVVFMQAHENLFQGEMLKYSAFATRVMINALFLEVWYHKRSPEALQDVVTEYKLRLAL
NCU06503.1	SPAPTFMVAHDNLFQGETLTYSAFGTRVMINALFLEVWYHKRSPEALQDVVTEYKLRLAL
FG05399.1 	STTVTFMEAHDNLFQGETLRYSTFGTRVMINALFLEVWYHKRSPEALQDVVTEYKLRLAL
AN9538.1  	STTMTVREAHDCLFQGDQTRYSAFATRVLINALFLQVWNHKRSFEALQDVVTEYKLRLAL
          	* : ..  **: ****:   **:*.***:******:** **** ****************

MG00373.1 	ETWEKSLELCEPEAVAVPLSAPHKGHPLLFNAKAMFRNARSRLEVDLKAVQEALRYHDSY
NCU06503.1	ETWEKSLEFCEHETVAAPLHTPQTGHPLIFNAKAMYRNARARLEVDLKTVQEALRYHDSY
FG05399.1 	ETWEKSVDMCETEAFPVPISAPHKGHPLIFNAKAMYRNARARLEVDLKAVQEALRYHEPY
AN9538.1  	ETWENSLEVCEPETIVVPLSTPQNGHPLIFNSMAVYRNTRARLEVDLKSIQEALRYHSSY
          	****:*::.** *:. .*: :*:.****:**: *::**:*:*******::*******..*

MG00373.1 	EVAAAMSSARDKVKRTTEMLKVIQECFNCIETAVLQGVRWVARTSPTQWSIEHPLCGLDM
NCU06503.1	EVAAAMSNARDRVRRSGEMIKVIQVCYDCIETAVDTGVRWVARTSPTNWSIEHPLGGMDL
FG05399.1 	EVAAAMTHARDRVKRSSEMVKVIDECYNCLETVVSQGVRWIARVSPTNWSVEHALCGMDL
AN9538.1  	EVAAAMTVAREKVKRSQEMNKVIQSCFECIEIAAIQGINWVAKTSATNWSVEHPLCGLDL
          	******: **::*:*: ** ***: *::*:* ..  *:.*:*:.*.*:**:**.* *:*:

MG00373.1 	MIILSLWLYRLEHDEEPATDDELAMYNKIRQLFDKDLDESYVAHLSSIVARLWGSMLDEV
NCU06503.1	MIILSLWLYRLEHDEEQATEEEQVMYKRVRELFARGLQEKIDGHLGAVVARLWASMLGEV
FG05399.1 	MIILSLWLYRLEHDEEPATQEELAMYNKVRQLLVKEIDENYMSQLSAVVARLWGSILDEV
AN9538.1  	MVILSLWLYRLEHDEEPASEAEMAIYNKVRNLFDDDAVDSCG-KLSSTVARVWGNILDGV
          	*:************** *:: * .:*:::*:*:     :.   :*.: ***:*..:*. *

MG00373.1 	VVWG2ITRLMGESFRLHSQALVGYVDDVEASSNVSTP-EMSQG-ADEDSVY
NCU06503.1	VVWG2ITRVMADSFRLHEQALIGYVDDIEASPSVTTPSMTSQG-ADEDSVY
FG05399.1 	VVWG2MTRLMGDSFKLHSQALVGYVDDPEASSNVSTPSMISQG-ADEDSVY
AN9538.1  	VVWG2ITKLMGESFKLHSQALVGYEDSLRVAKDQPIHAVPTKSLASVGTAY
          	**** :*::*.:**:**.***:** *. ..: . .  :  ::. *. .:.*
```
